# The impact of the digital economy on low-carbon innovation in the Yangtze River Delta region

**DOI:** 10.1371/journal.pone.0293835

**Published:** 2023-11-03

**Authors:** Xiaoli Wu, An Pan

**Affiliations:** 1 Business School, Shaoxing University, Shaoxing, Zhejiang, China; 2 School of Economics, Zhongnan University of Economics and Law, Wuhan, Hubei, China; Qufu Normal University, CHINA

## Abstract

This study narrows its focus to the Yangtze River Delta, an important region in China known for its advancements in both digital economy and low-carbon technology. In contrast to previous studies, we also examine the heterogeneous effects between central and non-central cities, as well as the role of local financial development, when analyzing the impact of the digital economy on low-carbon innovation. Based on the data of 41 cities from 2011 to 2019, we find a significant direct promoting effect of the digital economy on low-carbon innovation. Furthermore, the development of the digital economy indirectly enhances low-carbon innovation through local financial development. The heterogeneous analysis reveals a positive impact of the digital economy on low-carbon innovation in both central and non-central cities, with a stronger effect observed in non-central cities. These findings suggest several policy recommendations, including promoting digital economy and finance, green finance, and fostering regional integration in the Yangtze River Delta.

## Introduction

The report of the 20th National Congress of the Communist Party of China stresses the need to speed up digital economic growth and integrate it with the real economy. Moreover, the 14th Five-Year Plan of China prioritizes speeding up digital development and establishing a digital China. This includes leveraging the potential of data elements and building a strong digital economy, digital society, and digital government. These strategic orientations highlight the significant role of digital transformation in overall planning. Simultaneously, green innovation has emerged as an inevitable trend for economic and social development [1]. The abovementioned reports highlight the importance of creating a market-oriented green innovation system and taking steps to encourage green innovation. Xu et al. (2023) also emphasize the importance of green innovation as a crucial driver for achieving the "dual-carbon" goal and promoting high-quality development [2].

It is evident that, in line with the principles of China’s New Development Philosophy, both the digital economy and green innovation play crucial roles in driving economic transformation and promoting high-quality development. The combination and mutual reinforcement of these two sectors have become an important focus in the current era. This topic has commanded significant attention from the academic community, particularly regarding the potential of the digital economy, which utilizes digitized knowledge and information as production factors, in driving greening and de-carbonization efforts.

The Yangtze River Delta region is not only a vibrant hub for the development of the digital economy but also the most concentrated area for low-carbon pilot cities. In terms of the digital economy, the Yangtze River Delta region is the most vibrant and dynamic in China. The "*2022 Yangtze River Delta Digital Economy Development Report*" confirms that in the Yangtze River Delta, the digital economy has emerged as a driving force behind the rapid development of the overall economy and society. In 2021, the GDP of the Yangtze River Delta region reached 27.6 trillion yuan, accounting for 34.1% of the national total, with the digital economy accounting for 28% of the national total [3]. In terms of low-carbon innovation, according to the "*2023 Global Green and Low-Carbon Technology Patent Statistical Analysis Report*", the Yangtze River Delta had 10,737 green and low-carbon patent authorizations in 2022, far ahead of other major regions such as Beijing-Tianjin-Hebei (5,484 patents) and Guangdong-Hong Kong-Macao (4,934 patents). Since 2020, the number of invention patents in the Yangtze River Delta region has also been increasing rapidly, with an average annual growth rate of 24.5% [4].

After analyzing the aforementioned development situation, it is evident that the Yangtze River Delta region is at the forefront in terms of the development of the digital economy and low-carbon innovation. This prompts us to raise several research questions: Does the digital economy in this representative region of the Yangtze River Delta have a positive impact on low-carbon innovation? Are there any urban heterogeneity characteristics about these impacts? What are the specific mechanisms underlying these impacts? To answer these questions, we utilize panel data from 41 cities in the Yangtze River Delta from 2011 to 2019, to examine the effect of the digital economy on low-carbon innovation. Additionally, we conduct an analysis of the heterogeneous effect, categorized based on central and non-central cities. Furthermore, employing a mediation effect model, we examine how the digital economy affects low-carbon innovation through regional financial development.

Overall, focusing on the representative region of the Yangtze River Delta, it is crucial to delve into how the digital economy influences low-carbon innovation. This research will contribute to explaining certain development phenomena observed in the Yangtze River Delta. The contents are as follows: Section 2 reviews the previous related papers about our research topic. Section 3 analyzes the theoretical background and research hypothesis. Section 4 explains the research methodology. Section 5 provides the empirical results, discusses the reasons, and conducts the robustness test of regression results. Section 6 includes the conclusions, policy implications, limitations, and future research.

## Literature review

The global stage currently witnesses a rapid surge in the development of the digital economy, accompanied by the emergence and flourishing of new technologies like 5G, artificial intelligence (AI), etc. These advancements present crucial opportunities for the promotion of low-carbon development, and at the same time, they have made the progress of high technology and green development a research hotspot [5–7]. The existing literature primarily emphasizes the relationship between the digital economy and low-carbon development, with a particular focus on three aspects:

(1) Some studies treat innovation or low-carbon innovation as a vital intermediary and then explore the influence of the digital economy on various aspects. For example, some scholars have found that the digital economy can impact various aspects of the economy, including high-quality economic development or green economy [8–11], energy supply chain efficiency [12], carbon intensity [13], and optimization of industrial structure [14, 15], through facilitating innovation or low-carbon innovation. Although the aforementioned studies consider innovation as an intermediary variable, these studies confirm that the digital economy has a positive influence on low-carbon innovation.

(2) Some literature directly analyzed the impact of the digital economy on innovation or low-carbon innovation. For example, Dai et al. (2022) based on the provincial panel data, and found that the digital economy can effectively promote regional green innovation capability. The causal relationship is mainly realized through scientific research funds and human resources [16]. Meanwhile, some studies are based on data at the city level in China, analyzing the relationship between the digital economy and green innovation [17, 18]. Other studies are based on data at the enterprise level to analyze the relationship between these two factors [19–22]. In addition, scholars have also focused on the impact of an important component within the digital economy, namely digital finance, on green innovation [23, 24].

(3) Various studies have been conducted specifically analyzing the digital economy and low-carbon innovation in the Yangtze River Delta region. However, there is a dearth of research that establishes a correlation between these two factors. Regarding the digital economy, scholars have analyzed the impact of the digital economy in the Yangtze River Delta region on various aspects, such as urbanization [25], carbon emissions [26, 27], industrial structure [28], and green total factor productivity [29]. In the field of low-carbon innovation, several studies have examined the spatial network relationships about low-carbon innovation in the Yangtze River Delta region [30, 31]. Meanwhile, scholars have analyzed the impact of factors such as integration policies [32] and environmental regulations [33] on low-carbon innovation.

It is worth noting that there have been a few studies specifically focusing on the Yangtze River Delta region, and analyzing the impact of the digital economy on low-carbon innovation. For example, Lu (2022) [34] utilized relevant spatial econometric models to analyze the spatiotemporal evolution of the digital economy in the Yangtze River Delta region and its driving and spatial effects on green innovation [34]. Hu (2023) utilized the 2020–2022 regional innovation ecological index and digital index for the Yangtze River Delta region, along with a spatial econometric model, to find that the digital level significantly improves the innovation ecological level [35].

In short, the existing literature offers valuable insights and methodologies on the topic. However, certain areas present opportunities for further exploration. Firstly, most studies in this field tend to analyze the broader context of China, there is a lack of detailed and in-depth research specifically focusing on the representative Yangtze River Delta region. Secondly, few studies analyze the impact of digital finance, which is a component of the digital economy, on innovation. However, there is a lack of literature considering regional financial development as a mediating variable to understand how the digital economy influences low-carbon innovation. Thirdly, there is a lack of literature that differentiates between central and non-central cities in the Yangtze River Delta region, to analyze the heterogeneity characteristics of cities in terms of the impact of the digital economy on low-carbon innovation.

Building on the above-mentioned research gaps, this paper aims to expand on three aspects. First, this study specifically focuses on the representative region of the Yangtze River Delta and examines the impact of the digital economy on low-carbon innovation using panel data at the city level. Additionally, regional financial development is considered as a mediating variable, to analyze the direct and indirect mechanisms through which the digital economy affects low-carbon innovation. Moreover, the cities are divided into central and non-central cities to examine the heterogeneous effects.

## Theoretical background and research hypothesis

### Direct effects

The term "digital economy" originated from Don Tapscott’s book " The Digital Economy: Promise and Peril in the Age of Networked Intelligence " published in 1994 [36]. Since then, the concept of the digital economy has gained attention, but it remains undefined and covers a wide range of areas. According to the "14th Five-Year Plan for Digital Economy Development," the digital economy is considered the primary form of economy following agricultural and industrial economies. It is defined as an economy that is characterized by data resources as a key element, modern information networks as the main carrier, the integration and application of information and communication technology, and an important driving force of comprehensive digital transformation. This article further refines the concept of the digital economy into three aspects: digital technologies, modern information networks, and data resources. In this article, the empowering effects of the digital economy on low-carbon innovation will be elaborated in detail from these three aspects.

(1) In terms of digital technologies, the efficiency and cost advantages brought by digitalization have become increasingly prominent. They have been widely applied in the field of low-carbon technologies, facilitating the continuous integration and advancement of digital technologies and low-carbon innovation. For instance, the adoption of digital technologies like artificial intelligence and cloud computing is widespread in the energy and manufacturing sectors. This adoption greatly contributes to the digitization of energy systems and speeds up the innovation of low-carbon and zero-carbon energy technologies. (2) In the aspect of modern information networks, digital infrastructure plays a crucial role. The improvement of digital technologies, combined with the dissemination of digital information through network infrastructure, has reduced communication and replication costs compared to traditional information transmission. As accessing external knowledge and advanced experiences is an important way for innovation entities to enhance their innovation capabilities [37], the digital infrastructure eliminates barriers to information transfer, enhances the dissemination of knowledge, and strengthens the spillover effect of knowledge. Therefore, this will be beneficial for improving low-carbon innovation capabilities. (3) In terms of data resources, data elements penetrate the entire production process and chain, supporting full-chain low-carbon development. The full utilization of data resources can address the uncertainties, traceability, narrow scope, and ecological fragility faced in practical development, facilitating the formation of a low-carbon innovation system for innovative entities. Based on the discussion mentioned above, this study proposes the following research hypothesis:

Hypothesis 1: The digital economy has a significant direct promoting effect on low-carbon innovation.

### Indirect effects

Low-carbon innovation faces certain financial constraints. The reasons behind this are as follows: when companies engage in innovation activities, they often face high risks and uncertainties in returns due to the long innovation output cycles [38]. Compared to traditional technology innovation, low-carbon innovation requires larger upfront capital investment, has longer profit cycles, and involves risks that are more difficult to estimate [39]. Therefore, the realization of low-carbon innovation must be supplemented with a certain level of financial support.

As digital technologies such as big data, blockchain, and artificial intelligence become increasingly popular in the financial sector, the digital economy is actively facilitating the digitization of the finance industry. The rapid development of digital finance can significantly reduce financial transaction costs and enhance the accessibility of financial services, thereby better serving technological innovation. Specifically, in traditional financial services, the lack of transparency in information between enterprises and financial institutions leads to serious information asymmetry problems. This makes it difficult for enterprises to obtain financial support from financial institutions, which hinders the development of innovation activities. Previous research has also confirmed that when companies face financing constraints, it reduces their willingness to innovate and decreases their investment in innovation [40]. With the rapid development of the digital economy, the internet has become an important platform and medium for the disclosure of information by an increasing number of enterprises, making their information and behavior more transparent than before. Financial institutions can quickly grasp relevant information about enterprises through information platforms such as the Internet, gain a better understanding of their business capabilities, and identify enterprises with outstanding innovation abilities. This effectively mitigates the information asymmetry problem between financial institutions and enterprises, reduces the service barriers of financial institutions to enterprises, and improves the financial supply capacity of financial institutions [41, 42]. Ultimately, this reduces the financing difficulties faced by innovative enterprises caused by risk aversion and fills the funding gap for innovation activities. In conclusion, the digital economy has the potential to facilitate the advancement of the financial industry by enhancing the transparency of enterprise information and mitigating information asymmetry between financial institutions and enterprises. This, in turn, alleviates financing constraints experienced by enterprises and promotes their low-carbon innovation initiatives. Building upon this, the present study proposes the following research hypothesis:

Hypothesis 2: The digital economy can indirectly enhance the level of low-carbon innovation by empowering regional financial development.

### Heterogeneity of the impact effects

Due to variations in regional resource endowments, there exist certain differences in the development of the digital economy and innovation capabilities among different cities. On the one hand, digital economy competitiveness varies across regions due to differences in digital technology, market size, and human resources. In China’s eastern regions, the favorable market environment and digital infrastructure construction have facilitated the integration and development of the digital economy and physical economy, resulting in economies of scale. However, in the central and western regions of China, there is a slower pace of digital economy development compared to the eastern regions due to inadequate institutions, infrastructure, talent, and funding [43]. On the other hand, in major urban agglomerations such as the Yangtze River Delta, Pearl River Delta, and Beijing-Tianjin-Hebei region, there are significant disparities in innovation capabilities among different cities, leading to an uneven distribution of regional innovation outputs, inputs, and resources [44, 45]. There is significant regional heterogeneity in both the digital economy and low-carbon innovation.

The Yangtze River Delta region, as a highly integrated and economically developed world-class city cluster in China, displays regional heterogeneity among its sub-regions due to variations in factor endowments, economic development levels, and infrastructure construction. According to the "*Outline of Development Plan for Yangtze River Delta Regional Integration*", the cities in the Yangtze River Delta region are categorized into central and non-central areas. Specifically, the central areas include Shanghai, Nanjing, Hangzhou, Hefei, Suzhou, and other 27 cities, which enjoy certain advantages in terms of geographical location, human resources, and infrastructure construction. As a result, fiscal policies and other relevant measures tend to prioritize the central areas, leading to a relatively higher level of development in the digital economy and low-carbon innovation in these areas. Based on these observations, this study proposes the following research hypothesis:

Hypothesis 3: There are heterogeneous characteristics in the impact of the digital economy on low-carbon innovation between central and non-central cities in the Yangtze River Delta region.

## Research methodology

### Empirical model

To test the existence of the direct effect suggested by Hypothesis 1, we first construct the following baseline regression model:

$${Lcinno}_{it}=\beta_{0}+\beta_{1}{Dige}_{it}+\sum_{k=1}^{K}\beta_{k}X_{it}^{k}+\delta_{i}+\gamma_{t}+\varepsilon_{it}$$ (1)

where *Lcinno*_*it*_ represents the level of low-carbon innovation in city *i* during period *t*, *Dige*_*it*_ represents the level of digital economy development in city *i* during period *t*, *X* represents a set of control variables, *δ*_*i*_ represents city-specific fixed effects that remain constant over time, *γ*_*t*_ represents time fixed effects, and *ε*_*it*_ represents the error term.

To further explore how the digital economy influences low-carbon innovation, we employ a recursive equation to identify the mechanism through which the digital economy affects the ability of innovation in the Yangtze River Delta region. Specifically, referring to the study by Zhang et al. (2022) [46], we construct the following regression model building upon the model (1) to test whether financial development plays an intermediary role:

$${Fina}_{it}=\alpha_{0}+\alpha_{1}{Dige}_{it}+\sum_{k=1}^{K}\alpha_{k}X_{it}^{k}+\delta_{i}+\gamma_{t}+\varepsilon_{it}$$ (2)

$${Lcinno}_{it}=x_{0}+x_{1}{Fina}_{it}+x_{2}{Dige}_{it}+\sum_{k=1}^{K}x_{k}X_{it}^{k}+\delta_{i}+\gamma_{t}+\varepsilon_{it}$$ (3)

The specific testing steps are as follows: Under the condition that the regression coefficient of the baseline model if the regression coefficient of *Dige*_*it*_ on *Lcinno*_*it*_ is significant, we construct the following two regression models: the regression model (2) of *Dige*_*it*_ to *Fina*_*it*_ and the regression model (3) of *Dige*_*it*_ and *Fina*_*it*_ to *Lcinno*_*it*_. Further, based on the significance and magnitude of the estimated coefficients *α*_1_, *x*_1_, and *x*_2_ in the regression results, we evaluate whether financial development mediates the relationship between the digital economy and low-carbon innovation.

### Variables selection

#### Dependent variable

Low-carbon innovation (*Lcinno*_*it*_). Previous studies suggest that using low-carbon patent data provides a more objective and comprehensive measure of the level of low-carbon innovation. Therefore, following the approach of Pan et al. (2021) [47], this study uses the number of low-carbon patent applications as a proxy variable for low-carbon innovation.

#### Independent variable

Digital economy (*Dige*_*it*_). To comprehensively portray the level of the digital economy in cities, this study constructs an index evaluation system from two dimensions: internet development and digital inclusive finance. The entropy method is utilized for comprehensive estimation. The construction of indicators and measurement methods are as follows:

*(1) Index construction*. Existing research has generally employed two approaches to measure and assess the digital economy. The first approach is to measure the scale of the digital economy by identifying relevant industries [48, 49]. The second approach involves constructing a multidimensional indicator system to evaluate the level of the digital economy [50, 51]. Furthermore, Hu (2022) developed a National Digital Economy Competitiveness Index to systematically assess global digital competitiveness [52]. Zhang et al. (2023) utilized a three-stage DEA model and the Malmquist index to measure the digital economy output efficiency of various provinces and cities in China [53]. Based on existing research, this study aims to construct an evaluation indicator system for digital economy development at the city level, to objectively reflect the current state of digital economy development in the Yangtze River Delta cities.

Considering the limitations of city-level data, this study refers to the measurement indicators for the city-level digital economy constructed by Xu and Li (2020) [54]. The measurement indicators for the digital economy in the Yangtze River Delta region are constructed from two aspects: internet development and digital inclusive finance. Table 1 presents the specific indicators used. Internet development is the foundation of digital economy development. It will be measured using four sub-indicators: the number of broadband internet access users per 100 people, the number of mobile phone users per 100 people, the logarithm of total telecommunication service volume per capita, and the proportion of employment in information transmission, computer services, and software industries. This study adopts the digital inclusive finance index to represent the degree of integration of digital technology in traditional finance. It is worth noting that this index is derived from the research of Guo et al. (2020) [55] and calculated through a comprehensive indicator system, including three primary indicators: coverage breadth of digital inclusive finance, depth of use of digital inclusive finance, and degree of digitalization of digital inclusive finance, including 10 dimensions of indices.

**Table 1:** The index system of digital economy development level.

Index	First level indicators	Second level indicators	Unit	Directional attribute
Digital Economy Comprehensive Development Index	Internet development level	The number of broadband internet access users per 100 people	ten thousand households	+
		The number of mobile phone users per 100 people	ten thousand households	+
		The logarithm of total telecommunication service volume per capita	ten thousand households	+
		The logarithm of total telecommunication service volume per capita, and the proportion of employment in information transmission, computer services, and software industries	%	+
	Digital Inclusive Finance	Coverage breadth of digital inclusive finance	-	+
		Depth of use of digital inclusive finance	-	+
		Degree of digitalization of digital inclusive finance	-	+

Note: The coverage breadth of digital inclusive finance mainly includes indicators such as account penetration rate. The depth of use of digital inclusive finance mainly includes indicators related to payment services, money market fund services, credit services, insurance services, investment services, and credit services. The degree of digitalization of digital inclusive finance mainly includes indicators related to mobile access, affordability, credit access, and convenience.

*(2) Indicator measurement*. Existing research has used subjective weighting methods and objective weighting methods to determine the weights of various indicators in the digital economy. However, subjective weighting methods may be influenced by subjective biases when assigning weights to indicators, leading to measurement errors and an inadequate reflection of the overall situation. Therefore, this study utilizes the objective entropy weighting method, to measure the comprehensive development level of the digital economy in the Yangtze River Delta region. The specific measurement process is as follows:

First, standardize the indicators. In this study, all the indicators used are positive:

$$x_{ij}=\frac{x_{ij}-\min\{x_{j}\}}{\max\{x_{j}\}-\min\{x_{j}\}}$$ (4)

Second, determine the weight of the *j* indicator in the *i* year.:

$$\omega_{j}=\frac{x_{ij}}{\sum_{i=1}^{m}x_{ij}}$$ (5)

Third, calculate the information entropy of the *j* indicator.:

$$e_{j}=-\frac{1}{\ln\;m}\sum_{i=1}^{m}\omega_{ij}\times \ln(\omega_{ij}),S_{j}\in [0,1]$$ (6)

Fourth, calculate the redundancy of information entropy for the *j* indicator.:

$$d_{j}=1-e_{j}$$ (7)

Fifth, calculate the weight for the *j* indicator.:

$$\phi_{j}=\frac{d_{j}}{\sum_{j=1}^{m}d_{j}}$$ (8)

Finally, measure the level of digital economic development in various cities in the Yangtze River Delta region:

$$dige=\sum_{j=1}^{m}\phi_{j}\times \omega_{ij}$$ (9)

The computed final value represents the composite index of digital economic development in the Yangtze River Delta region, with a range between 0 and 1. A value approaching 1 indicates a higher level of digital economic development, while a value approaching 0 suggests a lower level.

#### Mediating variable

Based on the analysis of the theoretical mechanism, the mediating variable in this study is financial development (*Fina*_*it*_). The improvement of a region’s financial development level can alleviate the financing constraints faced by innovative activities of enterprises, thereby influencing the level of low-carbon innovation in that region. Therefore, this study selects the year-end outstanding loans of financial institutions in the city district as a proxy variable for financial development in the region.

#### Control variables

Drawing on relevant existing literature, this study selects several variables as control variables, including economic development level, industrial structure upgrading, local financial investment, and environmental regulations.

*(1) Economic development level (Pgdp)*. The economic development level reflects the degree of development. Regions with higher economic development have relatively more resources. For example, economically developed cities have better infrastructure, which can provide good support for low-carbon innovation activities in the region. Following the approach of most studies, this paper measures the economic development level of the region using per capita Gross Domestic Product (GDP).

*(2) Industrial structure upgrading (Instr)*. Enhancing a region’s financial development can alleviate financing constraints for innovative enterprises, directly influencing the level of low-carbon innovation in that area. Therefore, this paper represents the degree of industrial structure upgrading by the proportion of value added in the tertiary industry to GDP in the region.

*(3) Government financial investment (Gov)*. Local financial subsidies can provide funding compensation for enterprises engaged in innovation activities, which is an important influencing factor for such activities. Furthermore, the government’s fiscal revenues determine the extent of its financial subsidies. Therefore, this study selects the proportion of local government’s public fiscal revenues to the regional Gross Domestic Product (GDP) as a proxy variable for local financial investment.

*(4) Environmental regulations (Er)*. The intensity of environmental regulations can reflect the level of attention to environmental governance in a region, thereby affecting the pollution control costs of enterprises. The increasing strictness of environmental regulations can drive enterprises to engage in technological innovation and upgrading to reduce pollution control costs. Therefore, this paper measures the intensity of environmental regulations in a city using the comprehensive utilization rate of industrial solid waste.

### Sample and data

The level of digital economy development in the Yangtze River Delta is assessed using a range of indicators. In this study, the sample period selected is from 2011 to 2019 of 41 cities in the Yangtze River Delta, taking into account the calculation of inclusive finance indicators which commenced in 2011. Data on inclusive finance measures are sourced from the Digital Finance Research Center of Peking University and Ant Group, which jointly compiled the Digital Inclusive Finance Index [55]. Data on low-carbon patent grants, used to measure low-carbon innovation, is sourced from the IncoPat Global Patent Database. Data for the remaining secondary indicators, mediating variables, and other control variables are obtained from the "China Urban Statistical Yearbook". Additionally, natural logarithms are applied to low-carbon innovation, economic development level, and financial development variables to maintain consistency in terms of scale and measurement in this study.

Table 2 displays the descriptive statistics of each variable, indicating a significant disparity in the level of digital economy development among cities in the Yangtze River Delta. Specifically, among the 41 cities, the maximum value of the comprehensive digital economy index is as high as 0.943, close to 1, while the minimum value is only 0.0192. The standard deviation is 0.204, suggesting a substantial variation in the digital economy development level across the cities in the Yangtze River Delta. Furthermore, to eliminate the issue of multi-collinearity among variables, this study conducted correlation coefficient estimation and variance inflation factor tests on all variables. The test results indicate the absence of multi-collinearity problems.

**Table 2:** Descriptive statistics of variables.

Variable	Obs	Mean	Std.Dev.	Min	Med	Max
lnlcinno	369	5.350	1.400	1.790	5.280	8.390
dige	369	0.281	0.204	0.0192	0.205	0.943
lnPgdp	369	11	0.603	9.220	11	12.20
instr	369	0.437	0.0835	0.234	0.439	0.727
gov	369	0.167	0.0631	0.0760	0.150	0.356
er	369	0.924	0.0847	0.401	0.950	1

## Results and analysis

### Results of the baseline model

Table 3 displays the baseline regression findings, illustrating the influence of the digital economy in the Yangtze River Delta region on low-carbon innovation in cities. Column (1) represents the fixed effects regression results without controlling for any variables, while columns (2) to (5) display the fixed effects regression results after sequentially adding control variables. The results consistently show that the estimated coefficient of the core explanatory variable (*Dige*_*it*_) is significantly positive. These findings provide evidence that the advancement of the digital economy in the Yangtze River Delta region has a significant positive impact on enhancing the level of low-carbon innovation in cities, supporting Hypothesis 1.

**Table 3:** Results of the baseline model.

Variable	Dependent variable: lnlcinno				
	(1)	(2)	(3)	(4)	(5)
dige	2.523***	2.654***	2.729***	2.804***	2.808***
	(4.621)	(4.735)	(4.806)	(5.139)	(5.190)
lnPgdp		-0.256	-0.226	0.011	0.009
		(-1.203)	(-1.220)	(0.044)	(0.036)
instr			2.309*	2.581**	2.496**
			(2.003)	(2.378)	(2.160)
gov				5.575***	5.569***
				(3.971)	(3.968)
er					0.161
					(0.486)
cons	3.433***	6.107***	4.889**	1.414	1.320
	(14.385)	(2.742)	(2.305)	(0.497)	(0.480)
FE	Yes	Yes	Yes	Yes	Yes
Obs	369	369	369	369	369
Adjusted R^2^	0.738	0.739	0.742	0.759	0.759

Note: *, **, and*** denote significance at the 10%, 5%, and 1% significance levels, robust standard error is reported in parentheses. The symbols in the following table are the same as in this table.

Reasons, why the Yangtze River Delta’s digital economy can have a positive impact on low-carbon innovation, are as follows: On one hand, the Yangtze River Delta region has implemented a series of policy measures to promote the integration and synergy of the digital economy and low-carbon innovation. For example, in July 2022, the Yangtze River Delta region released a policy document titled "*Several Measures to Accelerate the Development of the Digital Economy and Promote Pilot Initiatives in the Ecological Green Integrated Development Demonstration Zone in the Yangtze River Delta*" [56]. This document includes measures such as integrating computational resources across industries and strengthening the green and resource-efficient construction of data centers. These policies provide a policy foundation for the two to achieve mutual integration and generate a "multiplier" effect.

On the other hand, in digital transformation, companies in the Yangtze River Delta are using digital tools to facilitate the low-carbon transformation of traditional industries. According to the "*Enterprise Digital Transformation Maturity Development Report (2022)*" [57], enterprises in the Yangtze River Delta region have the highest digital transformation maturity index of 26.7, which is 5.1% higher than the national average. Enterprises in various sectors, including industry, energy, transportation, and others, are embracing digital transformation. The goal is to drive low-carbon innovation and enable a shift towards more efficient and environmentally friendly production methods. Additionally, scholars also have highlighted the numerous advantages associated with the development of the digital economy. These advantages include enhancing data openness and sharing in the value chain, reducing information asymmetry, and facilitating the efficient allocation of innovation resources. Consequently, the digital economy plays a crucial role in promoting the innovation of low-carbon technologies [11, 22, 58].

The results regarding the control variables show that the estimated coefficients for industrial structure upgrading and fiscal investment are both significantly positive. This indicates that the advancement of industrial structure and increased fiscal investment in the cities of the Yangtze River Delta are conducive to the improvement of low-carbon innovation levels. However, the regression coefficients for economic development level and environmental regulations are not significantly positive. This suggests that the economic development level and environmental regulations in the cities of the Yangtze River Delta do not have a significant impact on low-carbon innovation.

### Robustness test

To ensure the robustness of the baseline regression results, this study conducts a robustness test by replacing the core explanatory variables and the dependent variables and further utilizes instrumental variable analysis to test for potential endogeneity issues in the model.

### Replacement of dependent and core explanatory variables

This study first conducts a robustness test by replacing the dependent variable. Specifically, the number of low-carbon patents granted is used as a substitute for the number of low-carbon patent applications to represent the level of low-carbon innovation in the cities of the Yangtze River Delta region. The regression results using this substituted variable are shown in columns (1) and (2) of Table 4.

**Table 4:** Results of the robustness test and endogeneity test.

Variable	Robustness test				Endogeneity test
	Replacement of Dependent Variables: lnlcinno1		Replacement of Core Explanatory Variables: lnlcinno		lnlcinno
	(1)	(2)	(3)	(4)	(5)
dige	0.893**	0.889**			
	(2.170)	(2.281)			
dige1			2.045**	1.961**	
			(2.490)	(2.309)	
dige_t-1_					5.565***
					(5.452)
lnPgdp		0.400*		0.138	-0.193
		(1.899)		(0.588)	(-0.908)
instr		0.460		1.926	2.850***
		(0.382)		(1.665)	(2.770)
gov		4.464**		5.263***	5.775***
		(2.225)		(3.546)	(4.862)
er		0.758**		0.192	0.197
		(2.045)		(0.555)	(0.592)
FE	Yes	Yes	Yes	Yes	Yes
Obs	369	369	369	369	369
Adjusted R^2^	0.441	0.474	0.723	0.739	0.700
Kleibergen-Paap rk LM					21.244[0.0000]
Kleibergen-Paap rk Wald F					28.378{16.38}

Note: The values within [] represent the P-value, while the values within {} represent the critical values for the Stock-Yogo weak identification test at a 10% significance level.

Next, we replace the core explanatory variable that measures the digital economy. Following the approach of Bai and Zhang (2022) [59], three additional secondary indicators of the digital economy are included, and their measurements are recalibrated using entropy values. The regression results using these recalibrated variables are presented in columns (3) and (4) of Table 4.

It is important to note the following regarding the aforementioned results: columns (1) and (3) present the fixed-effects regression results without controlling for additional variables, while columns (2) and (4) present the fixed-effects regression results with the inclusion of control variables. The results indicate that, even after replacing the dependent variable and core explanatory variable, the signs and significance levels of the regression results remain consistent with the baseline regression results. This finding suggests a certain degree of robustness in the findings.

#### Endogeneity test

To address omitted variable bias and potential reverse causality between the digital economy and low-carbon innovation, this study employs the lagged one-period digital economy as an instrumental variable for the digital economy. Column (5) in Table 4 reports the results of the 2SLS regression using the lagged one-period digital economy as an instrumental variable. The Kleibergen-Paap rk LM statistic is 21.224, with a corresponding p-value of 0.000, indicating a significant rejection of the null hypothesis of "insufficient instrument strength". The value of the Kleibergen-Paap rk Wald F statistic is 28.378, which is greater than the critical value of 16.38 at the 10% level, suggesting the absence of weak instrument problems.

In conclusion, after carefully considering the endogeneity issue in our study, the regression coefficient and significance of the core explanatory variable, the digital economy, remain consistent with the baseline model’s regression results. This further confirms the robustness of the regression findings in this study.

### Mechanism analysis

To further examine whether the development of the digital economy indirectly enhances low-carbon innovation through empowering regional financial development in the Yangtze River Delta region, this study conducts empirical tests using the constructed mediation model (Eqs (2) and (3)). The regression results are presented in Table 5.

**Table 5:** Results of mechanism analysis: Mediating effect.

Variable	lnlcinno	lnfina	lnlcinno
	(1)	(2)	(3)
dige	2.808***	0.375***	2.481***
	(5.190)	(2.840)	(4.709)
lnfina			0.892***
			(3.148)
lnPgdp	0.009	0.344***	-0.299
	(0.036)	(2.763)	(-1.592)
instr	2.496**	0.335	2.327*
	(2.160)	(0.742)	(1.755)
gov	5.569***	-0.603	6.124***
	(3.968)	(-1.178)	(4.727)
er	0.161	0.169	0.002
	(0.486)	(0.963)	(0.007)
cons	1.320	3.423**	-1.774
	(0.480)	(2.483)	(-0.669)
FE	Yes	Yes	Yes
Obs	369	368	368
Adjusted R^2^	0.759	0.926	0.772

The regression results in column (2) demonstrate that the coefficient of the digital economy on financial development is significant at the 1% level, indicating that the development of the digital economy promotes local financial development. The results in column (3) show the regression results after including the mediation variable. It is observed that the coefficient of the digital economy on low-carbon innovation is significantly positive with a magnitude of 2.481. Compared to the regression results of Eq (1), the coefficient value decreases. This suggests that the development of the digital economy in the Yangtze River Delta region indirectly enhances low-carbon innovation levels by promoting local financial development. Therefore, Hypothesis 2 is confirmed.

The digital economy indirectly enhances low-carbon innovation levels by empowering regional financial development. This is due to the following reasons: Firstly, the development of advanced digital technologies, such as 5G, big data, and blockchain, is continuously improving the technological aspect of financial services. These technologies provide solid hardware support for promoting low-carbon innovative development. For example, financial innovation driven by digital technologies includes the establishment of green innovation funds, the provision of special green innovation subsidies, and the creation of integrated pilot zones. These financial measures offer flexible software support for the deep integration of digital finance and green innovation. Secondly, the digital economy promotes digital finance, in order to reduce transaction costs for low-carbon innovation. In the internet era, digital finance provides decision-makers with accurate investment and financing information [60]. It enables efficient matching of low-carbon innovation funds, accelerating financial flow into innovative areas [61]. This leads to lower search and financing costs for low-carbon innovation entities, ultimately promoting green innovation development. Thirdly, the development of the digital economy facilitates the scale effect of financial services, leading to an increase in investment in low-carbon innovation research. The digital economy pushes traditional financial services to integrate networking and intelligence [62]. This means that the influence of financial development on green innovation will be impacted by new information technologies like the Internet. With the continuous empowerment of the digital economy on financial development, the participation intensity of green innovation entities will increase, and the value of related financial services will grow exponentially. Thus, as the digital economy drives financial innovation, the enabling effect of low-carbon innovation will continue to strengthen.

### Heterogeneity analysis

Due to the differences in resource endowments and development stages among cities in the Yangtze River Delta region, it is necessary to explore the heterogeneous impact of the digital economy on low-carbon innovation in these cities. Based on the "Outline of the Development Plan for the Integrated Development of the Yangtze River Delta Region" issued by the State Council, this study divides cities in the Yangtze River Delta region into central area cities and non-central area cities to conduct regional heterogeneity regression analysis.

As evident from the regression results presented in Table 6, both central area cities and non-central area cities in the Yangtze River Delta region demonstrate a significant positive impact of the digital economy on levels of low-carbon innovation. Moreover, this promoting effect is found to be even stronger in non-central area cities. Hence, Hypothesis 3 is confirmed.

**Table 6:** Heterogeneity testing based on urban characteristics perspective.

Variable	Central cities	Non-central cities
	(1)	(2)
dige	2.957***	3.406***
	(3.969)	(3.114)
lnPgdp	0.047	0.161
	(0.124)	(0.340)
instr	2.433	2.933
	(1.372)	(1.011)
gov	6.313	5.956***
	(1.486)	(4.614)
er	0.369	-0.180
	(0.635)	(-0.355)
FE	Yes	Yes
Obs	369	369
Adjusted R^2^	0.751	0.769

The stronger positive influence of the digital economy on low-carbon innovation in non-central cities can be attributed to a few reasons. Firstly, central cities in the region have higher economic development, better infrastructure, and richer resources. This leads to a more mature development of the digital economy and low-carbon innovation, with limited potential for improvement in these areas. According to the "*2022 Report on the Development of the Digital Economy in the Yangtze River Delta*" published by the China Industrial Information Security Development Research Center [63], cities such as Shanghai, Hangzhou, Nanjing, and Suzhou are at the forefront of the digital industrialization in China. Suzhou, Ningbo, Wuxi, and other cities have a comparative advantage in industrial digitalization. Overall, the central cities in the region are leading in the development of the digital economy in China, but face relatively greater difficulties in further improvement, resulting in a relatively smaller role in promoting low-carbon innovation. In contrast, non-central cities are experiencing high-growth phases in the digital economy and low-carbon innovation. As a result, the digital economy has a stronger positive impact on low-carbon innovation in non-central cities.

## Conclusion and policy recommendations

The digital economy provides new possibilities for low-carbon development, and the study of the relationship between the digital economy and low-carbon innovation has become a current research hotspot. However, the Yangtze River Delta region in China is a frontrunner in the development of both the digital economy and low-carbon innovation, yet there is limited literature that specifically focuses on the impact mechanism between these two aspects in this representative region. This study determined how the digital economy affects low-carbon innovation in the Yangtze River Delta, taking into consideration the mediating role of regional financial development, as well as the heterogeneity between central and non-central cities. Therefore, based on the panel data of 41 cities in the Yangtze River Delta region from 2011 to 2019, this study employs fixed effects model and mediation effects model to examine the impact of the digital economy on low-carbon innovation. Additionally, we analyze the mediating mechanisms and city heterogeneity characteristics in the aforementioned impact process.

The conclusions drawn from the study are as follows: The development of the digital economy in the Yangtze River Delta significantly promotes the enhancement of urban low-carbon innovation levels. Additionally, financial development plays a significant mediating role in the process by which the digital economy fosters low-carbon innovation. After dividing the Yangtze River Delta region into central and non-central cities, it is observed that the digital economy has a significant positive impact on low-carbon innovation levels in both types of cities, with a stronger promoting effect observed in non-central cities.

Based on the above conclusions, the following recommendations are proposed: (i) accelerate the high-quality development of the digital economy in the Yangtze River Delta region and promote the integration of digitization and low-carbon innovation. The region should leverage the advancements in the digital economy and focus on establishing innovative development models for industrial digitization and digital industrialization. This will enhance the role of the digital economy in driving low-carbon innovation effectively. (ii) Accelerate the development of digital finance and green finance in the Yangtze River Delta region. This includes the promotion of green credit, green insurance, and carbon finance. There is a need to actively foster mechanisms that facilitate the integration of green and digital finance. This will enable the financial sector to fully leverage its intermediary role and enhance the empowerment effect of the digital economy on low-carbon innovation. (iii) Enhance the awareness of regional integration in the Yangtze River Delta and emphasize top-level design and regional coordination. The empirical conclusions suggest that there are differences in the development levels of the digital economy and low-carbon innovation among different cities. Therefore, it is necessary to accelerate the coordinated development of the digital economy among cities in the Yangtze River Delta region, in order to promote collaborative low-carbon innovation among different regions.

There are a few limitations of the current study, on the one hand, low-carbon innovation has not been subdivided into specific industries, so empirical analysis in different industries has not been conducted yet. On the other hand, due to limitations in accessing relevant research data, case studies on specific industries or companies have not been conducted yet. For future studies, considering the significant differences in digital transformation and low-carbon innovation across different industries, it is important to differentiate between various industries for empirical analysis. Moreover, it is recommended to choose a representative case study from Hangzhou, which is the most developed area in the Yangtze River Delta in terms of digital economy. This approach will allow for a comprehensive analysis of the impact of the digital economy on low-carbon innovation in the region.
